# The Role of Exosomes in Offspring Metabolic Programming in Gestational Diabetes: Mechanisms and Potential Applications

**DOI:** 10.7150/ijbs.131080

**Published:** 2026-03-30

**Authors:** Jing Zhou, Dongmei Wang, Miao Yu, Xiao Zhai, Yingna Song, Jieying Liu, Xinhua Xiao

**Affiliations:** 1 NHC Key Laboratory of Endocrinology (Peking Union Medical College Hospital), Diabetes Research Center of Chinese Academy of Medical Sciences, Department of Endocrinology, Peking Union Medical College Hospital, Chinese Academy of Medical Sciences & Peking Union Medical College, Beijing, 100730, China.; 2 Department of Endocrinology, Genetics, Metabolism, Beijing Children's Hospital, Capital Medical University, National Center for Children's Health, Beijing, China.; 3 Department of Obstetrics and Gynaecology, Peking Union Medical College Hospital, Chinese Academy of Medical Sciences & Peking Union Medical College, Beijing, China.; 4 Center for Biomarker Discovery and Validation, National Infrastructures for Translational Medicine (PUMCH), Institute of Clinical Medicine, Peking Union Medical College Hospital, Chinese Academy of Medical Sciences & Peking Union Medical College, Beijing, China.

**Keywords:** Gestational diabetes mellitus, Exosomes, Maternal-fetal communication, Offspring, Glucose and lipid metabolism

## Abstract

Genetic predisposition and unhealthy lifestyles are well-known contributors to disorders of glucose and lipid metabolism, including type 2 diabetes, obesity, and metabolic dysfunction-associated fatty liver disease. However, these factors alone cannot fully explain the rapidly rising prevalence of these conditions. Emerging evidence highlights the pivotal role of the intrauterine environment in gestational diabetes mellitus (GDM) in shaping epigenetic modifications and metabolic reprogramming, thereby predisposing offspring to long-term metabolic complications. Exosomes have recently been identified as key mediators of maternal-fetal communication. In GDM, both the quantity and cargo (e.g., proteins, miRNAs) of exosomes are altered. These altered exosomes not only contribute to maternal glucose and lipid metabolic abnormalities but also act as a critical vector for transmitting adverse metabolic signals to the offspring. This exosome-mediated communication disrupts placental function and the development of fetal metabolic organs, ultimately programming the offspring for long-term metabolic disorders. In this review, we summarize the characteristic changes of maternal exosomes in GDM and explore the potential mechanism by which exosomes regulate offspring metabolism during maternal-fetal crosstalk. We also propose the possible direction of exosomes in application, providing insights into early-life strategies for the prevention of metabolic diseases.

## 1. Introduction

Gestational diabetes mellitus (GDM) is one of the most common complications in pregnancy 1. The latest data from the International Diabetes Federation reported that one fetus in six live births is born to GDM mothers 2. A substantial body of evidence suggests that GDM has the potential to permanently alter metabolic programming and physiology, thus contributing to metabolic disturbances in both mothers and their offspring 3.

The early intrauterine environment can affect the metabolic status in later life, a concept known as the developmental origins of health and disease (DOHaD) theory 4. Since 1986, numerous studies have underscored the pivotal role of the intrauterine period in cellular growth, differentiation, and organogenesis, showing that maternal and early-life exposures are essential for normal fetal growth and the long-term development of offspring 5-8. The potential mechanisms underlying the effects of adverse intrauterine environment, such as hyperglycemia in GDM, on metabolism during pregnancy and offspring metabolism include abnormal placental function 9, epigenetic and metabolic reprogramming 10. However, the relevant mechanisms are not completely clear, and early intervention measures still need further research. In this context, increasing attention has been directed toward soluble and vesicle-mediated signaling pathways that can cross the placental barrier and deliver regulatory molecules from the mother to the fetus, among which exosomes have emerged as particularly promising candidates.

As a class of extracellular vesicles (EVs), exosomes can stably carry miRNAs, mRNAs, proteins, and lipids in biofluids 11. These cargo-enriched exosomes are now recognized as critical mediators of intercellular communication, thereby directly modulating gene expression and signaling in recipient cells 12. *In vivo* and *in vitro* studies indicate that blocking exosome biogenesis or release can attenuate inter-tissue signal transmission (e.g., adipose-derived circulating miRNAs that regulate the liver and muscle), suggesting that exosomes may play causal or amplifying roles in metabolic programming 13-15.

Pregnancy itself is a state of dynamic intercellular communication, during which both the concentration and molecular profile of circulating exosomes are remodeled 16. Moreover, the characteristic pathological milieu of GDM can markedly alter the quantity, cargo composition, and biological function of exosomes derived from maternal tissues 17-19. These GDM-associated changes may represent a key pathway through which adverse metabolic effects are transmitted to the offspring. Indeed, studies in mouse models have demonstrated that maternal exosomes can cross the placenta and enter the fetal circulation 20. Consequently, exosomes derived from GDM mothers can modulate placental function and key aspects of fetal metabolic programming, such as metabolic organ maturation, fetal growth, and body composition 21-24. Furthermore, exosomes are easily detectable in blood and can serve as potential targets for therapeutic intervention. Therefore, considering exosomes as a measurable and potentially modifiable mode of interorgan communication can complement and deepen our understanding of GDM-maternal-fetal interactions and guide potential early diagnostic and interventional strategies.

In this review, we summarize the characteristic changes of maternal exosomes under GDM, explore the transgenerational role of exosomes in offspring metabolism during maternal-fetal crosstalk, and highlight their potential therapeutic applications. These insights may advance our understanding of the pathogenesis and early intervention during the critical therapeutic window for metabolic disorders.

## 2. Overview of Exosomes in Pregnancy

EVs are lipid bilayer-enclosed particles actively secreted by cells, originating from various compartments, including endosomes and the plasma membrane. They are widely distributed in bodily fluids such as blood, saliva, and urine, as well as in cell culture supernatants 11. According to the biological pathway, size and release mechanism, EVs can be divided into three main types: exosomes (30-150 nm, classified as small EVs (sEVs), derived from the fusion of multivesicular bodies (MVBs) and plasma membrane), microvesicles (100-1000 nm, produced by outward budding of plasma membrane) and apoptotic bodies (1000-5000 nm, formed in the process of programmed cell death) 25-27. EVs transport diverse functional molecules, including proteins, lipids, RNA/DNA species, and bioactive enzymes 28, with release occurring across nearly all cell types 29. This ubiquitous distribution underscores their crucial role in cellular communication mechanisms.

### 2.1 Exosome biogenesis, uptake, and characterization

**Figure 1:**
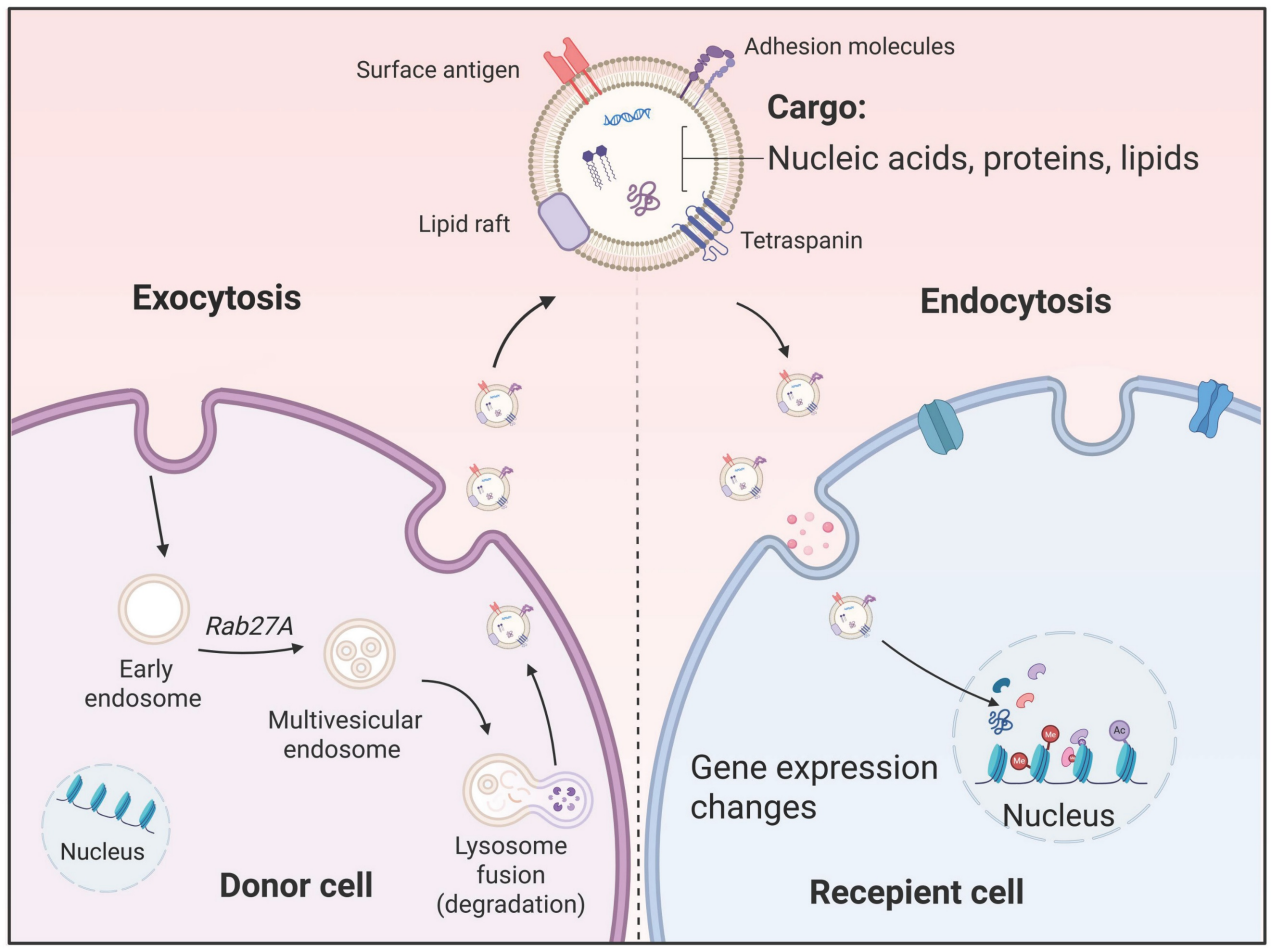
** The biological generation and uptake of exosomes.** The biological generation of exosomes begins with the invagination of the cell membrane forming an early endosome. Upon maturation into a late endosome, the membrane undergoes internal budding to encapsulate cytoplasmic contents, creating a polycentric vesicle body packed with numerous intracellular vesicles. Ultimately, this polycentric vesicle body fuses with the cell membrane, releasing its vesicles into the extracellular environment, thus becoming exosomes. Through fluid circulation, exosomes deliver their cargo to local or distant target cells via membrane fusion, endocytosis, or ligand-receptor interactions.

As a crucial subcategory of EVs, exosomes serve as vital messengers in both physiological and pathological processes. The biological generation of exosomes begins with the invagination of the cell membrane, forming an early endosome. Upon maturation into a late endosome, the membrane undergoes internal budding to encapsulate cytoplasmic contents, creating an MVB packed with numerous intracellular vesicles 30. Ultimately, this MVB fuses with the cell membrane, releasing its vesicles into the extracellular environment, thus becoming exosomes 31. The lipid bilayer of exosomes effectively shields and protects their cargo from degradation. Through bodily fluid circulation, exosomes are transported to local or distant target cells, where they deliver their cargo via membrane fusion, endocytosis, or ligand-receptor interactions 27. These molecular messages subsequently modulate the function of recipient cells, playing critical roles in physiological processes and pathological conditions 32, 33 (**Figure 1**).

Currently, exosomes are commonly isolated from blood (including plasma and serum), urine, and cell culture conditioned medium 18, 34. Standard isolation techniques include differential (ultra)centrifugation, density gradient centrifugation, size-exclusion chromatography, and others 35. To enhance the yield, these methods are often combined with ultrafiltration for concentration. The purified exosomes are then characterized using techniques like electron microscopy and nanoparticle tracking analysis (NTA) to confirm their size and morphology. Furthermore, the presence of characteristic exosomal surface markers (e.g., CD63, CD81, CD9, TSG101) is typically verified by western blot or flow cytometry 36. Due to their critical role in pathogenesis, along with their high abundance and detectable composition, exosomes and their cargo have emerged as a highly promising source of biomarkers for disease diagnosis and prevention 37.

### 2.2 Exosome transport in pregnancy

**Figure 2:**
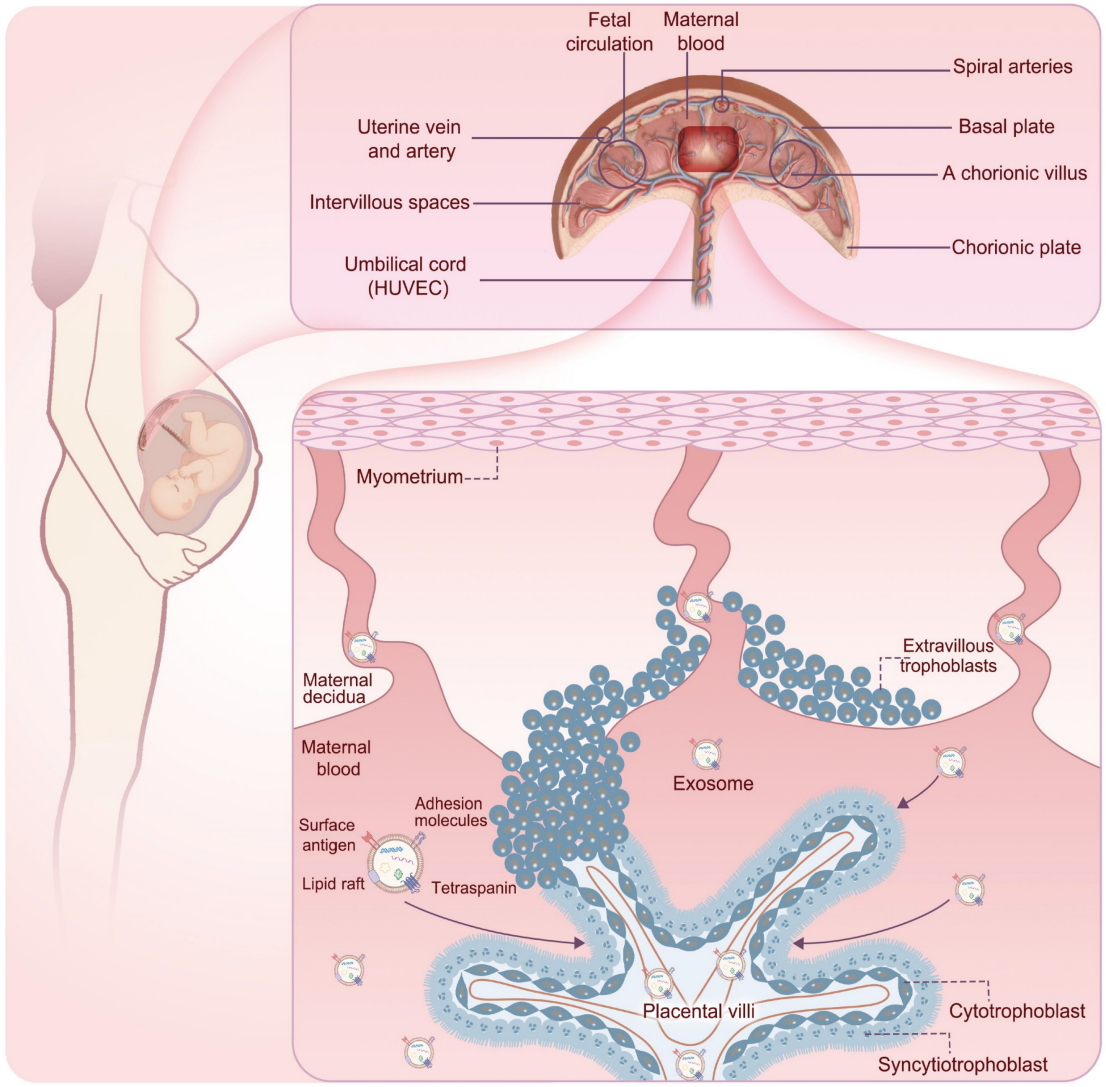
** The process of exosomes crossing the placenta into the fetus.** Circulating maternal exosomes first encounter the apical surface of the syncytiotrophoblast, the outermost cell layer of the chorionic villi. At this interface, exosomes are internalized predominantly via active endocytic pathways. Once inside trophoblast cells, exosomes enter the endosomal system, where their cargos may be integrated into placental signaling networks or sorted into vesicles destined for basolateral exocytosis. Through this transcytotic route, intact exosomes and their released cargos can be released into the villous stroma and fetal capillary compartment, thereby reaching the fetal circulation.

During pregnancy, circulating exosome concentrations in healthy pregnant women increase approximately 20- to 50-fold compared to those in non-pregnant women and continue to increase throughout gestation 16, 38. This range reflects differences in isolation methods, quantification techniques, and gestational timing across studies. These vesicles contribute to processes such as embryo implantation, placental function, and modulation of the maternal immune system 39, demonstrating their regulatory role in cell-cell communication. Furthermore, the placental barrier is not a static wall but a dynamic interface where maternal exosomes can be selectively transferred to the fetal side 40-42. Circulating maternal exosomes first encounter the apical surface of the syncytiotrophoblast, the outermost cell layer of the chorionic villi. At this interface, exosomes bind to trophoblast membranes through adhesion molecules and receptor-ligand interactions involving, for example, tetraspanins, integrins, and heparan sulfate proteoglycans, and are internalized predominantly via active endocytic pathways, including clathrin- and caveolin-dependent endocytosis, macropinocytosis, and phagocytosis 41, 43. Once inside trophoblast cells, exosomes enter the endosomal system, where their cargos may be integrated into placental signaling networks or sorted into vesicles destined for basolateral exocytosis. Through this transcytotic route, intact exosomes and their released cargos can be released into the villous stroma and fetal capillary compartment, thereby reaching the fetal circulation 41, 43. Experimental mouse models using fluorescently labeled or genetically engineered sEVs have provided direct *in vivo* evidence that vesicles administered on the maternal side can cross the placenta, be detected in fetal tissues, and induce functional changes, demonstrating maternal-fetal transfer of exosomal cargo across the placental barrier 20, 44. Fetal endothelial and parenchymal cells, such as hepatocytes 45, pancreatic β cells 46, and adipocytes 47, can further take up these vesicles via similar receptor-mediated and endocytic mechanisms, allowing maternal exosomes to deliver regulatory nucleic acids and proteins to fetal target tissues and potentially modify gene expression, epigenetic marks, and metabolic pathways that contribute to fetal metabolic programming 44 (**Figure 2**).

## 3. Exosomes in GDM

In GDM, the characteristic pathological milieu—hyperglycemia, insulin resistance, oxidative stress, and low-grade inflammation—profoundly alters exosome secretion by the placenta, adipose tissue, endothelial cells, and other maternal tissues 17-19. Specifically, hyperglycemia and insulin resistance, as key pathological stimuli, activate signaling pathways such as PKC/NF-κB and HIF-1α in placental trophoblast cells, upregulating the expression of exosome biogenesis-related proteins (e.g., Rab27a), thereby significantly increasing the release of placental-derived exosomes 48, 49. Meanwhile, in states of insulin resistance and lipotoxicity, endoplasmic reticulum stress and local inflammatory responses are also activated in adipose tissue, promoting the secretion of exosomes carrying specific miRNAs and adipokines by adipocytes and macrophages 50, 51. Endothelial EVs show reduced vasoprotective miRNAs and increased procoagulant cargos, thereby leading to vascular dysfunction 52, 53. As a result, exosomes display characteristic changes in abundance, cargo composition, and bioactivity, which are thought to represent a key route through which adverse metabolic signals are transmitted from mother to offspring 17-19. Circulating exosomes in women with GDM show higher concentrations and larger mean diameters than those in normoglycemic pregnant women, and their levels correlate positively with maternal glycemia 17. Functionally, injections of exosomes isolated from the plasma of GDM mothers into mice reduce basal insulin signaling and insulin responsiveness in skeletal muscle 54, 55.

To further elucidate the roles of these vesicles, miRNA sequencing and proteomic analyses have been widely used to identify differentially expressed cargos in GDM-associated exosomes 56, 57, many of which are closely linked to pathways involved in glucose and lipid metabolism and metabolic inflammation. In the following section, we discuss the exosomes in GDM according to their major molecular cargos.

### 3.1 miRNAs in exosomes from GDM

Among the compositional changes in exosomes, altered miRNA profiles are the most striking. These miRNAs, as key post-transcriptional regulatory factors, are considered major mediators of the metabolic disturbances conveyed by GDM-derived exosomes 58, 59. Several studies have reported that GDM induces significant changes in exosomal miRNA levels 58-60. For example, compared with those in the normal glucose tolerance (NGT) group, 9 miRNAs were significantly upregulated and 14 downregulated in exosomes isolated from the GDM group, and the levels of these miRNAs were consistent in the placenta, circulating exosomes, and skeletal muscle 58. These significantly altered exosomal miRNAs are positively correlated with fasting plasma glucose (FPG) and OGTT levels in GDM mothers 61, 62. Other studies have reported that significantly altered miRNAs in exosomes from GDM mothers include miR-122-5p 60, miR-92a-3p 63, miR-320b 64, miR-99-5p 65, etc. These miRNAs are transported to target cells and regulate insulin signaling, adipogenesis, placental function, angiogenesis, and other pathways (**Table 1**).

#### 3.1.1 Blood-derived exosomal miRNAs in GDM

Given their accessibility and dynamic reflection of systemic metabolic changes, blood-derived exosomes have been extensively characterized in the context of GDM. As a highly abundant liver-associated miRNA, miR-122-5p is central in regulating lipid and glucose metabolism 66. In metabolic disorders, miR-122-5p is frequently observed at abnormally elevated levels 66, 67. miR-122-5p may affect gluconeogenesis by targeting Glucose 6 phosphatase 68, and also promote hepatic lipid synthesis through regulating the genes including Acly, Mttp, and Srebp-1 69. Consistent with its metabolic functions, multiple studies have reported significant alterations of miR-122-5p in GDM exosomes 59, 60, 65. However, the reported directions of change are inconsistent. For instance, one study reported upregulation of miR-122-5p in GDM serum exosomes during 6-15 gestational weeks (GW) 59, whereas Shen Y *et al.* found that the expression of miR-122-5p was decreased in plasma exosomes from GDM mothers at 8-12 GW, followed by an increase in late gestation 60. Another study found that the expression of miR-122-5p in plasma exosomes was downregulated in GDM mothers during 10-16 GW, suggesting a link to subsequent energy metabolism disorders and insulin resistance 65. These discrepancies may reflect differences in gestational age at sampling, biofluid type (serum vs. plasma), exosome isolation protocols, and detection methods. Future work is needed to reconcile these inconsistencies and clarify the precise role of exosomal miR-122-5p in GDM.

miR-92a-3p has been confirmed to play a crucial role in GDM. Compared with healthy pregnant women, women with GDM exhibit significantly elevated levels of miR-92a expression in peripheral blood 70. In plasma exosomes from GDM mothers, miR-92a-3p expression was also significantly increased in mid-pregnancy but markedly decreased in late pregnancy 63, 68, a trend may be influenced by gestational age. In function, miR-92a can target KLF2 to increase insulin secretion in β cells 71, and induces suppressor of cytokine signaling 2 (SOCS2) and inhibits the expression of NOS2 in skeletal muscle cells to prevent hyperglycemia by increasing glucose uptake 58, 63. In addition, as an angiogenesis-related miRNA, the upregulation of miR-92a-3p in GDM may affect placental vascular remodeling and maternal-fetal interface function 72, which is associated with the risk of complications such as placental hemodynamic disorders and macrosomia 73.

The miR-29 family serves as a dual regulator of insulin secretion and signaling. Exosomes secreted by pancreatic β-cells are rich in miR-29, which targets the regulatory subunit p85α of PI3K, leading to insulin resistance 74. In the liver, miR-29 promotes lipid synthesis by directly targeting Sirt1 and Foxo3 75. Placenta-derived exosomes extracted from the plasma of GDM mothers showed significantly elevated levels of both miR-29a-3p and miR-29b-3p, suggesting that the placenta may regulate maternal metabolism through exosomal secretion 59. In addition, miR-29 can target DNA methyltransferase (DNMT) and participate in epigenetic regulation 59, which may be related to the long-term effects of metabolic programming.

#### 3.1.2 Urine-derived exosomal miRNAs in GDM

Current research on GDM-derived exosomes has predominantly focused on those isolated from blood and placental tissues, with comparatively less attention given to urinary exosomes. This discrepancy is likely attributable to technical challenges in purification and the fact that urine composition is more susceptible to confounding factors such as diet and exercise 76, 77. Despite these challenges, Herrera-Van Oostdam AS *et al.* isolated placenta-derived exosomes (PLAP+) from the urine of GDM mothers, revealing that miR-516-5p, miR-517-3p, and miR-518-5p levels were significantly elevated from the second trimester through the third trimester compared to healthy controls 78. Notably, their shared target genes are predominantly expressed in placental and adipose tissues, suggesting these exosomes may reach these tissues, and placental regulation constitutes an integral component of maternal-fetal communication 78.

In summary, most differentially expressed exosomal miRNAs in GDM are involved in glucose and lipid metabolic pathways, and many show promise as early biomarkers that could improve the prediction and diagnosis of GDM 60, 65. However, changes in miRNA expression levels may be inconsistent across studies, likely due to small sample sizes, different gestational weeks, lack of pre-pregnancy or early-pregnancy glycemic data, and heterogeneity in clinical diagnostic criteria for GDM. In addition, most studies focused on the effects of exosomes on the GDM mothers. In fact, these exosomes may cross the placental barrier and influence fetal development and metabolism, a possibility that warrants more in-depth investigation in future studies. Furthermore, research on the differential effects of exosomes derived from various subtypes or severities of GDM remains limited, highlighting another critical avenue for future investigation.

### 3.2 Proteins in exosomes from GDM

Proteomic approaches have been widely applied to characterize the cargo of GDM-derived exosomes, revealing a large number of differentially expressed proteins. These proteins are associated with inflammatory responses 57, cellular stress 79, maternal metabolism, and changes in placental function 80 (**Table 2**).

Exosome proteomics revealed the potential role of the complement system and coagulation factors in the pathogenesis of GDM 81. In serum exosomes from GDM patients, reduced expression of C3, C5, C4B, C4BPB, and C4BPA suggests suppression of the classical complement/antigen-binding pathway, which may be related to insulin resistance 79. The upregulation of C7, C9 (key components of the membrane attack complex), and F12 (part of the contact system/endogenous coagulation pathway) in GDM exosomes suggests that the complement terminal pathway and associated inflammatory and coagulation processes are abnormally activated 79. Such an imbalance may promote inflammatory damage in the placental microenvironment and endothelial dysfunction, potentially contributing to preeclampsia and macrosomia 82.

Differentially expressed proteins in the exosomes reflect maternal metabolism and placental functional status. Thrombospondin 1 (Thbs1) protein was highly expressed in adipose-derived exosomes of GDM mice 18. It interacts with CD36 and Tgfβ receptors, activates Tgfβ/Smad2 signaling pathways, ultimately inducing insulin resistance 18. The expression of calcium-regulated heat-stable protein 1 (CARHSP1) was downregulated in the placental exosomes of GDM mothers, which may increase gluconeogenesis and affect inflammatory signal transduction, leading to a hyperglycemic state 83, 84. Other differentially expressed proteins, such as ACPP (which is related to prostaglandin metabolism), CRYZL1, and ANO10, have also been identified, although their precise roles in GDM remain to be elucidated 83.

Together, these data suggest that exosomal proteins in GDM not only reflect maternal metabolic stress and placental dysfunction but may also participate in maternal-fetal communication by carrying protein signals across or modulating the placental barrier, thereby contributing to metabolic programming in the offspring. Moreover, the distinct proteomic signatures of GDM-associated exosomes highlight their potential as minimally invasive biomarkers and as targets for interventions aimed at improving the intrauterine environment.

### 3.3 Other non-classical contents in the exosomes from GDM

In addition to miRNAs, exosomes derived from GDM pregnancies also carry other classes of non-coding RNAs, including circular RNAs (circRNAs) and long non-coding RNAs (lncRNAs), which have emerged as potential contributors to disease pathology and biomarkers of GDM (**Table 3**). The expression of hsa_circRNA_0039480 in plasma exosomes of GDM mothers was higher than that in the NGT group at different stages, and was positively correlated with OGTT in the mid-trimester 61. The lncRNA AC006064.4, which is highly expressed in GDM exosomes, can be used as a predictor of GDM-related macrosomia 85. Both circRNAs and lncRNAs may function as "molecular sponges" for miRNAs, thereby indirectly regulating the activity of miRNA, so as to participate in regulating metabolic pathways 86. Nevertheless, research on the underlying mechanisms of these lncRNAs and circRNAs is poorly understood, highlighting the need for more in-depth studies.

Furthermore, changes in the maternal microbiome may be responsible for metabolic diseases 87, 88. Microbial components in exosomes can regulate the progression of GDM. Through microbiome analysis, Chang CJ *et al.* found that the ratio of *Firmicutes/Bacteroidetes* in serum sEVs of GDM mothers increased, which is associated with insulin resistance 89. The relative abundance of *Bacteroidetes* decreased, which resulted in reduced butyrate production, reduced insulin sensitivity, and increased inflammation 89, 90. The enrichment of Gram-negative pathogens in GDM exosomes may lead to metabolic endotoxemia and activation of the inflammatory response to aggravate the inflammatory response, damage the insulin signaling mechanism, and eventually lead to hyperglycemia 89.

In summary, exosomes originating from GDM carry diverse classes of bioactive molecules, including miRNAs, proteins, other non-coding RNAs, and microbiome-related components. These molecules not only mirror maternal metabolic stress, placental dysfunction, and inflammatory status, but also may act as active mediators of maternal-fetal communication by modulating insulin signaling, lipid metabolism, vascular remodeling, and epigenetic programming in target tissues. The multifaceted exosomal signatures observed in GDM therefore provide a mechanistic link between the adverse maternal environment and offspring metabolic risk, while at the same time offering a rich source of minimally invasive biomarkers and potential targets for early intervention.

## 4. The Role of Exosomes in Offspring Metabolism

The DOHaD hypothesis proposes that an adverse intrauterine environment can shape long-term metabolic health in the offspring, largely through epigenetic modifications. Multiple clinical studies indicate that the metabolic effects of GDM on offspring begin as early as infancy and can persist into adulthood 91-94. However, how signals are transmitted across the placenta to offspring and induce epigenetic changes remains unclear.

**Figure 3:**
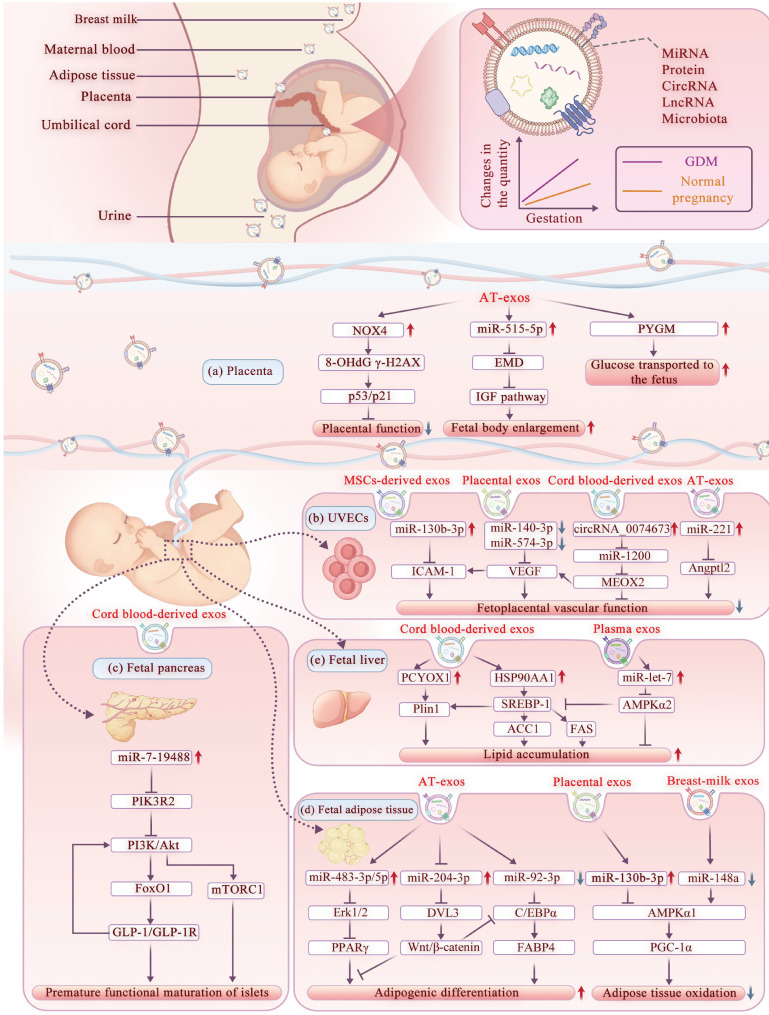
** The mechanism of GDM exosomes affecting offspring metabolism.** Maternal exosomes in GDM exert multiple effects on the offspring metabolism. These exosomes enter the fetus through the placenta and then disrupt placental function via trophoblast cells, regulate fetal growth through umbilical vein endothelial cells, and modulate glucose and lipid metabolism by impacting the pancreas, adipose tissue, and liver. Abbreviations: AT-exos: Adipose Tissue-derived Exosomes; CEBPα: CCAAT/Enhancer Binding Protein α; DVL3: Dishevelled Segment Polarity Protein 3; Erk1/2: Extracellular Signal-Regulated Kinases 1 and 2; FABP4: Fatty Acid Binding Protein 4; FAS: Fatty Acid Synthase; FoxO1: Forkhead Box Protein O1; GDM: Gestational Diabetes Mellitus; GLP-1/GLP-1R: Glucagon-like Peptide-1 / GLP-1 Receptor; HSP90AA1: Heat Shock Protein 90 Alpha Family Class A Member 1; ICAM-1: Intercellular Adhesion Molecule 1; IGF pathway: Insulin-like Growth Factor pathway; MEOX2: Mesenchyme Homeobox 2; mTORC1: Mechanistic Target of Rapamycin Complex 1; NOX4: NADPH Oxidase 4; PCYOX1: Prenylcysteine Oxidase 1; PGC-1α: Peroxisome Proliferator-activated Receptor Gamma Coactivator 1-α; PI3K: Phosphoinositide 3-Kinase; PIK3R2: Phosphoinositide-3-Kinase Regulatory Subunit 2; Plin1: Perilipin 1; PPARγ: Peroxisome Proliferator-activated Receptor γ; PYGM: Muscle Glycogen Phosphorylase; SREBP-1: Sterol Regulatory Element-binding Protein 1; UVECs: Umbilical Vein Endothelial Cells; VEGF: Vascular Endothelial Growth Factor; Wnt/β-catenin: Wingless-related Integration site /β-catenin.

In this context, exosomes have attracted growing attention as molecular carriers capable of transporting maternal bioactive cargos to the fetus, thereby modulating epigenetic and signaling pathways and exerting more profound and long-lasting effects on offspring metabolism 56, 95, 96. Given the selective recognition and internalization of exosomes by placental trophoblast cells, the pathogenic exosomes in GDM mothers can directly deliver the abnormal molecular signals to the fetus, influencing the growth, development, and metabolism of the offspring 17, 22, 97, 98. On the other hand, upon sensing abnormal maternal metabolic cues the placenta itself also secretes exosomes into the fetal circulation, thereby amplifying or transducing the maternal metabolic stress to the developing fetus 43, 99, 100. In this section, we further explored the role of exosomes in regulating offspring metabolism during maternal-fetal communication, organized by their actions on different target organs and cell types (**Figure 3**).

### 4.1 Targeting the placenta

The placenta is a critical interface between maternal and fetal circulation, facilitating molecular exchanges via exosomes 101, 102. In metabolic diseases, normal placental development is disrupted, increasing the risk of adverse pregnancy outcomes 31, 103. Exosomes from GDM mothers can directly act on placental trophoblasts, regulating the gene expression of oxidative stress 98, glucose, and lipid transport proteins 104, thus affecting fetal growth and development 105.

Specifically, the adipose tissue of obese mothers secretes exosomes rich in NADPH oxidase 4 (NOX4), which can be transported to trophoblast cells, upregulate the levels of 8-OHdG and γ-H2AX, activate the P53/P21 axis, resulting in severe oxidative DNA damage, premature placental aging, and adverse pregnancy outcomes 98. Quantitatively, GDM adipose exosomes are 1.7 times more abundant than in healthy mothers and correlate positively with birth weight Z-score 104. This effect is linked to GDM exosomes boosting glycolysis genes (PGK2, GCK) and suppressing mitochondrial gene SUCLA2 in placental cells, indicating that the glycolytic pathway was enhanced and mitochondrial function was impaired 104, 106, 107. The increase in PYGM involved in glycogenolysis suggests an increased transfer of glucose to the fetus, which may lead to fetal overgrowth 104, 108. Similarly, injection of adipose tissue-derived EVs loaded with miR-515-5p into pregnant mice led to increased fetal body weight and blood glucose levels 105. miR-515-5p may enhance placental glucose uptake by targeting the EMD gene 105, which encodes the Emerin protein that regulates the IGF signaling pathway 109. These studies have demonstrated the potential role of GDM exosomes in regulating placental function, fetal growth, and metabolism.

### 4.2 Targeting the umbilical vein endothelial cells (UVECs)

In the placental villous structure, UVECs line the luminal interface of the fetal side circulation 110. As the core unit for material exchange between the fetus and the mother, its function and transported blood components reflect the fetal metabolic status to some degree 111. Primary HUVECs obtained from GDM women exhibited persistent inflammatory and oxidative phenotypes under normal glucose levels, and this “glucose memory effect” could increase metabolic disease risk in offspring through epigenetics 112, 113. In this process, the exosomes of GDM mothers can directly act on the UVECs. By modulating UVEC proliferation and migration, these exosomal signals may disrupt fetoplacental vascular function, thereby affecting fetal growth and development.

For instance, levels of miR-140-3p and miR-574-3p were reduced in placenta tissue-derived exosomes from GDM dams. This reduction offsets the inhibition of VEGF expression, resulting in abnormal proliferation, migration, and tube formation of UVECs, which may ultimately impair fetal development 114. Furthermore, Gao Z *et al.* found that miR-130b-3p was elevated in human placental mesenchymal stem cells (MSCs)-derived exosomes, targeting ICAM-1 downregulation and inhibiting UVEC proliferation and angiogenesis 19. Furthermore, circRNA_0074673 was upregulated in umbilical cord blood exosomes from GDM mothers, where it sponged miR-1200 to upregulate MEOX2 115, which is associated with endothelial cell aging and migration 116, inhibiting endothelial cell migration and angiogenesis. Additionally, miR-221, which is enriched in exosomes from obese mice, inhibits endothelial cell proliferation and angiogenesis by downregulating Angptl2 expression via its 3' untranslated region, contributing to placental dysplasia during pregnancy, which impacts fetal development 117. However, current studies have primarily documented the immediate effects of these exosomes on UVECs. Their long-term consequences for offspring's health warrant further investigation.

### 4.3 Targeting the fetal pancreas

The human pancreas develops from the endoderm during early embryogenesis through the formation of pancreatic buds, branching morphogenesis, and cell differentiation, establishing a primitive framework with both endocrine and exocrine functions by late gestation 118, 119. However, critical regulatory mechanisms such as precise control of insulin secretion remain incomplete until after birth 120. This intricate process can be disrupted by maternal hyperglycemia, interfering with pancreatic development. Studies indicate significant changes in pancreatic diameter, pancreatic mass, and β-cell mass in the offspring of HFD dams after birth 121, 122. In fact, pancreatic remodeling may have occurred as early as the fetal period, and exosomes may play an important role in this process.

Zou L *et al.* demonstrated that exosomes administered to pregnant mice can cross the placenta to reach the fetal pancreas 22. Exosomes derived from umbilical cord blood of GDM mothers were shown to enhance the expression of GLP-1R, p-Akt, and p-S6 in fetal islet β cells of mice, while also increasing the expression of disallowed genes 22. This induces a state of precocious maturation in the fetal pancreas before birth. They further injected exosomes from the cord blood of GDM mothers into pregnant rats 46. These exosomes, which were enriched with miR-7-19488, targeted PIK3R2 mRNA in the pancreatic islets of the fetal rats 46, leading to p85β translation arrest and subsequent activation of PI3K to phosphorylate Akt 46. The PI3K/Akt pathway serves as a critical regulator of fetal growth 123. Its activation enhances FoxO1 and mTORC1 signaling pathways, promoting the development and maturation of α and β cells. This process also intensifies stimulation of the local GLP-1/GLP-1R axis 46. Consequently, these events drive the premature functional maturation of pancreatic islets during early prenatal stages, predisposing the offspring to metabolic diseases in later life 22.

### 4.4 Targeting the fetal adipose tissue

The body composition of the fetus may be determined as early as the first trimester when the gastrula forms 124. During the mid-trimester of pregnancy, adipogenesis begins. MSCs first develop into preadipocytes, which then proliferate and differentiate into adipocytes in the late trimester 125. This process will continue in several years after birth through hyperplasia and hypertrophy of adipocytes, which is highly plastic and can be programmed by the intrauterine environment, influencing the long-term metabolic health of offspring 126.

Maternal exosomes significantly influence fetal adipocyte differentiation, thereby modulating fetal growth and metabolic programming. This is evidenced by the differential expression of specific miRNAs in association with neonatal adiposity 127. For instance, plasma exosomal miR-483-3p is significantly elevated in mothers of obese neonates compared to those of lean neonates 127, which is associated with lipid accumulation and adipocyte differentiation in low birth weight adults 128, suggesting a persistent effect. Moreover, the level of miR-483-5p is markedly increased in the umbilical cord blood exosomes of obese neonates 127. This miRNA promotes adipogenesis in 3T3-L1 preadipocytes by suppressing the Erk1/2 signaling pathway 129, thereby increasing the risk of long-term metabolic disorders. Other miRNAs, including has-miR-204-3p and has-miR-15a, are also significantly altered in the cord blood exosomes of obese neonates 130, 131. These miRNAs have been demonstrated to regulate adipose differentiation and intramuscular fat synthesis 130, 131. Notably, has-miR-92a-3p and has-miR-181, which are associated with adipose differentiation and brown adipose tissue activity, are differentially expressed in umbilical cord blood of obese neonates, suggesting their potential role in the development of fetal adipose tissue 127.

Maternal exosomes play a significant role in modulating lipid synthesis and degradation in offspring. Studies have revealed an inverse correlation between birth weight and the expression levels of specific miRNAs 132. For instance, the downregulation of miR-130b-3p, miR-29a-3p, and miR-let-7a-5p in the placenta, are all associated with increased birth weight, suggesting a potential role for these miRNAs in regulating lipid metabolism of offspring 132. Jiang *et al.* demonstrated that high glucose conditions promote the secretion of exosomes enriched with miR-130b-3p from placental trophoblast cells. These exosomes suppress the expression of energy metabolism proteins, including AMPKα1 and PGC-1α 133, which is linked to impaired adipose tissue oxidation in the fetus 113. This metabolic disruption, if sustained, can easily lead to metabolic abnormalities in the offspring 113. Moreover, miRNA levels in breast milk exosomes have been correlated with body composition in infants. At 1 month of age, the abundance of miR-148a and miR-30b in breast milk exosomes was lowered in the infants born to obese mothers compared to controls 134. Reduced miR-148a levels may disrupt adipogenesis and insulin signaling by targeting AMPKα1, IGF-1, and the PI3K/AKT pathway, leading to increased fat mass in infants one month after birth 134. In contrast, miR-30b was positively correlated with infant weight and fat mass, with its overexpression stimulating lipogenesis and increasing lipid droplet size 134. Given the relatively immature gastrointestinal tract of newborns, efficient molecular communication between mother and infant via exosomal miRNAs may regulate infant growth and fat mass.

### 4.5 Targeting the fetal liver

During the fetal period, the liver primarily functions in hematopoiesis, and its metabolic development begins to activate rather than being activated only after birth 135. Recent studies have revealed that maternal high-fat diet can alter the expression of key genes involved in lipid and glucose metabolism in the fetal liver before birth through epigenetic mechanisms, leading to intrahepatic lipid accumulation 136-138. This early programmed alteration may be partially mediated by exosome-driven signaling, ultimately impairing the metabolic adaptation and reprogramming capacity in the liver of offspring when faced with postnatal nutritional transitions 139.

Díaz M *et al.*, found that the upregulation of both PCYOX1 and HSP90AA1 in exosomes derived from the umbilical cord blood of small-for-gestational-age (SGA) infants can predict hepatic adipose accumulation by the age of seven 45. Mechanistically, PCYOX1 is involved in degrading prenylated proteins, and PCYOX1-deficient mice on a high-fat diet exhibit reduced body weight and visceral fat 140. Similarly, HSP90AA1, a highly conserved molecular chaperone, promotes hepatic lipid accumulation in mice by modulating the activity of SREBP-1 141. Furthermore, let-7c-5p level was significantly reduced in plasma exosomes of GDM mothers during early pregnancy 49. Let-7c-5p regulates glucose metabolism by targeting IRS1, IRS2, forkhead box protein 1 (FOXO1), and PPAR 132. In the offspring of obese dams, increased miR-let-7 levels downregulated AMPKα2 in the liver, thereby disrupting early hepatic metabolism 142. However, due to the limited research on the role of exosomes in regulating fetal liver metabolic development, more studies are needed to further clarify the potential mechanism.

In summary, maternal exosomes in GDM exert multiple effects on the offspring. They disrupt placental function by targeting trophoblast cells, regulate fetal growth and development by acting on umbilical vein endothelial cells, and modulate glucose and lipid metabolism by influencing the development and function of the pancreas, adipose tissue, and liver. However, these studies primarily focus on the immediate effects of exosomes on the fetus, while their long-term metabolic impacts require further investigation. Additionally, these exosomes may influence fetal immune system programming, which in turn could affect offspring metabolism. Nevertheless, the role of exosomes in this specific process is still poorly understood. Unraveling these developmental dysregulations is critical, as they may have implications for metabolic health in the offspring, warranting in-depth investigation in future studies.

## 5. Application of Exosomes in Reducing Metabolic Diseases in Offspring

The evidence reviewed above demonstrates that maternal-derived exosomes exert programming effects on offspring metabolism. These mechanistic insights not only establish exosomes as key signaling vectors in mediating either pathogenic or protective effects, but also provide a robust biological rationale for their application in the early prediction and intervention of metabolic diseases in the offspring.

### 5.1 Early prediction using exosomal biomarkers

Changes in exosomal cargo mirror early molecular events in GDM and fetal metabolic programming, often preceding overt clinical phenotypes, which makes them attractive minimally invasive biomarkers 43. For example, plasma exosome hsa_cirRNA_0039480 is highly expressed in GDM patients at different stages, supporting its utility as an early detection biomarker 61. Early-pregnancy placenta-derived exosomes hsa-miR-3665 and hsa-miR-6727-5p levels varied with pancreas beta cell function in GDM, which have been implicated in placental dysfunction 143. Ye *et al.* further demonstrated that a panel of five plasma-derived exosomal miRNAs achieved an AUC of 0.82 for early GDM prediction 65. These examples illustrate how exosomal profiling can capture molecular perturbations before clinical manifestations appear, enabling earlier diagnosis and intervention.

### 5.2 Engineered exosomes and delivery strategies

Beyond diagnostics, exosomes are increasingly transitioning from passive biomarkers to active interventional tools. However, current studies largely focus on adult metabolic disease models, and the translational gap can be bridged by engineering strategies grounded in the intergenerational communication pathways reviewed above.

One straightforward approach is harnessing native exosomes from healthy sources. Based on the evidence that maternal exosomes from normal pregnancies actively improve offspring metabolic outcomes, several studies have administered placenta- or umbilical cord-derived exosomes from healthy donors to GDM mouse models. This approach has been shown to enhance maternal glucose tolerance and insulin sensitivity 22, and to ameliorate insulin resistance and obesity in the offspring 46, 144. Beyond these pregnancy-specific sources, stem cells represent another promising reservoir of therapeutic exosomes. For example, ADSC-derived exosomes promote M2 macrophage polarization and reduce white adipose tissue mass in obesity models 145. These studies suggest that replenishing “protective” exosomal signals may counteract the maladaptive programming induced by adverse intrauterine environments.

Given the key role of specific miRNAs and proteins in mediating maternal-offspring metabolic crosstalk, engineering exosomes to enrich these bioactive molecules is a rational step. Engineered exosomes have been explored in various conditions, including diabetes 146, obesity 147, and aging-related disorders 148. In the context of GDM, some exosomal miRNAs are significantly dysregulated, including miR-122-5p, miR-92a-3p, miR-29, etc. Given their potential to cross the placental barrier and influence fetal development, maternal administration of engineered exosomes loaded with these specific miRNAs may modulate offspring metabolism, an approach supported by emerging experimental evidence. For instance, injection of adipose tissue-derived EVs loaded with miR-515-5p into pregnant mice led to increased fetal body weight and blood glucose levels 105. Together, these findings support the feasibility of designing exosomes that carry defined miRNA payloads to intervene in specific pathways during critical developmental windows in offspring.

Beyond cargo modulation, engineering the surface of exosomes for targeted delivery is a forward-looking strategy. The natural tropism of placental exosomes for fetal organs offers a blueprint for targeted delivery 149. Future strategies may involve surface modification (e.g., with tissue-specific homing peptides) to enhance accumulation in offspring liver, pancreas, or hypothalamus 150, 151. Alternatively, hybrid nanovesicles that combine exosomal membrane proteins with synthetic carriers could offer a more scalable and biocompatible option 152, 153.

Collectively, these findings suggest that if substances (e.g., miRNA, proteins) that have significant changes in GDM or offspring can be constructed into engineered exosomes and applied in clinical therapy, this may provide new ideas for the prevention and treatment of GDM and offspring metabolic diseases 154.

## 6. Conclusion

**Figure 4:**
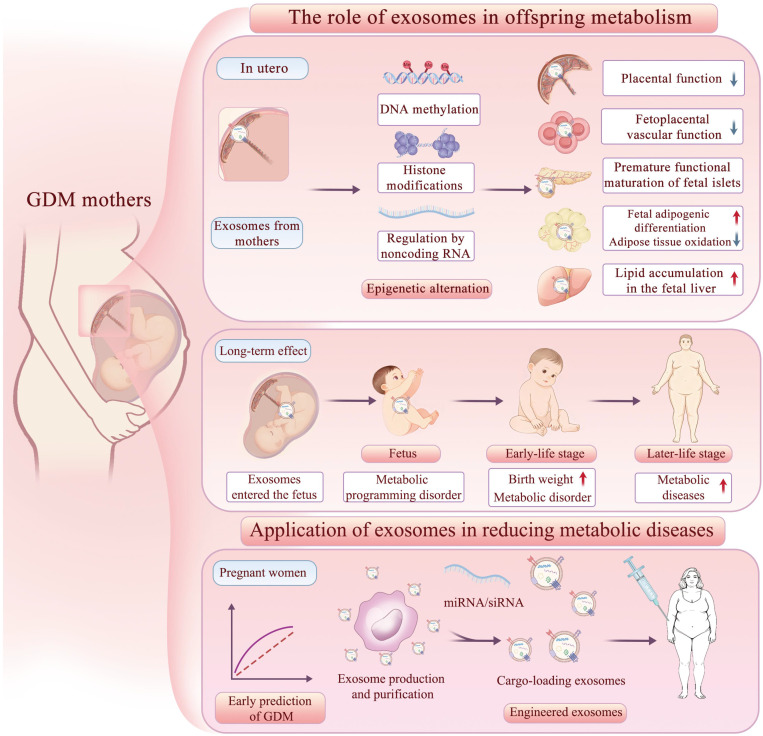
** The effects and applications of exosomes on offspring metabolism.** Exosomes from GDM mothers cross the placenta into the fetus, affecting placental function and the metabolic development of umbilical vein endothelial cells, fetal pancreas, adipose tissue, and liver through epigenetic mechanisms, thereby affecting the metabolic health of offspring in both short-term and long-term perspectives. MiRNAs in exosomes can serve as early diagnostic markers for GDM, and engineered exosome interventions may help reduce the incidence of metabolic diseases in the future.

In this review, we summarize—across miRNAs, proteins, other non-coding RNAs, and microbiome-related cargos—the characteristic changes of GDM-associated maternal exosomes in blood, placenta, adipose tissue and other sources, and relate them to their actions on the placenta, fetal metabolic organs, and long-term offspring phenotypes. To our knowledge, this is the first review to systematically connect maternal exosomal alterations in GDM with organ-targeted effects in the offspring, thereby providing an integrated view of how exosomes may transmit maternal metabolic stress across generations (**Figure 4**).

At the same time, our synthesis also reveals important limitations in the current literature. First, there is a striking lack of full “end-to-end” causal evidence that directly links: (i) the GDM state; (ii) defined changes in maternal exosomal cargo; (iii) exosome trafficking across the placenta and uptake by specific fetal organs; (iv) persistent metabolic alterations in the offspring. Most studies examine only one or two segments of this chain. Second, the cellular origins and trafficking routes of exosomes within the maternal-fetal interface remain incompletely defined. Although placenta- and cord blood-derived exosomes are frequently studied, it is often unclear whether they are of maternal, fetal, or placental origin. Third, most data remain correlative and short-term, focusing on exosomal signatures in pregnancy or early life, with relatively few studies linking maternal exosome profiles to metabolic outcomes in adolescence or adulthood. Finally, translation toward intervention is still preliminary: while engineered or “healthy pregnancy-derived” exosomes show promise in preclinical models, offspring-targeted delivery and safety, as well as standardized production and characterization, remain challenging.

Future research should therefore prioritize multi-level, longitudinal, and mechanistic designs. In humans, prospective pregnancy cohorts integrating maternal metabolic phenotyping with serial profiling of exosomal cargo and long-term follow-up of offspring metabolic outcomes will be essential to map temporal trajectories and identify robust, early biomarkers of risk. In parallel, causal studies in animal models are needed to manipulate exosomes along the entire maternal-placental-fetal axis—for example, by selectively altering exosome release in maternal adipose tissue or placenta, genetically or pharmacologically editing key exosomal cargos, or transferring well-defined exosome populations from GDM versus control mothers into naïve pregnancies, and then tracking offspring organ-specific metabolism. Advancing exosome-tracing technologies (e.g., genetic reporters, *in vivo* imaging, placental-on-a-chip and organoid models) will be critical to visualize exosome trafficking across the placental barrier and their uptake by fetal liver, pancreas, adipose tissue, skeletal muscle, and brain.

The clinical translation of engineered exosomes is accelerating, marked by the emergence of clinical studies that have reported preliminary safety and efficacy 155, 156. However, the application of these strategies in metabolic diseases remains immature. On the translational side, the therapeutic potential of exosomes in preventing offspring metabolic disease will depend on transitioning from observational administration to mechanism-based design. These mechanistic insights we reviewed above can be translated into engineering strategies: parental cell engineering to overexpress protective miRNAs implicated in metabolic programming; post-isolation loading of small molecules or siRNAs targeting key metabolic regulators; and exosome-inspired synthetic vesicles that replicate the functional properties of placental or milk exosomes while enabling production.

Future work should also focus on optimizing loading strategies for exosomes, and rigorously evaluating safety, dosing, and timing of administration during critical developmental windows. Standardization of exosome isolation, characterization, and nomenclature will also be required to enable cross-study comparison and clinical implementation. If these gaps can be addressed, exosome-based strategies-combining their biomarker value (capturing molecular events before overt phenotypes) with their therapeutic carrier function-may ultimately provide a novel avenue to reduce the long-term metabolic burden of GDM for both mothers and their children, transforming early-life prevention of metabolic diseases.

## Figures and Tables

**Table 1:** miRNAs in exosomes from GDM.

Contents in GDM exosomes	Source	Sample/Model	Trimester of pregnancy	Isolation of exosomes	Characterization of exosomes	Differential expression in GDM	Target/effect	Reference
miR-122-5p	Serum	Human (Caucasian/White)	6-15GW	exoRNeasy Serum/Plasma Maxi kit	TEM	↑	PPARα↓, Sirt1↓ in the liver (when miR-122-5p is upregulated)	59
	Plasma	Human (Asian)	10-16GW	Differential ultracentrifugation	TEM; NTA; WB	↓		59, 65
	Plasma	Human (Asian)	8-12GW	Ultracentrifugation	TEM; NTA; WB	↓		59, 60
miR-132-3p	Serum	Human (Caucasian/White)	6-15GW	exoRNeasy Serum/Plasma Maxi kit	TEM	↑	KLF7↓ in endothelial cells	59
miR-29a-3p	Serum	Human (Caucasian/White)	6-15GW	exoRNeasy Serum/Plasma Maxi kit	TEM	↑	Sirt1↓, Foxo3↓ in the liver	59, 75
miR-29b-3p	Serum	Human (Caucasian/White)	6-15GW	exoRNeasy Serum/Plasma Maxi kit	TEM	↑		59, 75
miR-182-3p	Serum	Human (Caucasian/White)	6-15GW	exoRNeasy Serum/Plasma Maxi kit	TEM	↑	INSR1↓ in the skeletal muscle	59, 157
miR-92a-3p	Plasma	Human (Caucasian/White)	<18GW; 22-28GW; 37-40GW	Ultracentrifugation	TEM; NTA; WB	<18GW ↑; 22-28GW↑; 37-40GW↓	SOCS2↑, NOS2↓ in skeletal muscle cells (when miR-92a-3p is upregulated)	63
miR-16-2-3p	Plasma	Human (Caucasian/White)	<18GW; 22-28GW; 37-40GW	Ultracentrifugation	TEM; NTA; WB	<18GW ↑; 22-28GW↑; 37-40GW↓	ACADM↓ in vascular cells (when miR-16-2-3p is upregulated)	63, 158
miR-16-5p	Urine (PLAP+)	Human (Caucasian/White)	8-20GW; 24-28GW; 32-39GW	Urine Exosome Purification kit	TEM; WB	8-20GW (-); 24-28GW↑; 32-39GW (-)	IRS1↓, IRS2↓ in the liver	78
miR-1299	Plasma	Human (Asian)	24-28GW	Differential ultracentrifugation	TEM; NTA; WB	↑	STAT3/FAM3A↓ in hepatocytes	159
miR-423-5p	Plasma	Human (Asian)	10-16GW	Differential ultracentrifugation	TEM; NTA; WB	↑	IGF1R↓, GYS1↓ in HepG2 cells	65
miR-148a-3p	Plasma	Human (Asian)	10-16GW	Differential ultracentrifugation	TEM; NTA; WB	↓	TGFB2↑, FGF2↑ in retinal microvascular endothelial cells	65, 160
miR-192-5p	Plasma	Human (Asian)	10-16GW	Differential ultracentrifugation	TEM; NTA; WB	↓	SCD-1↑ in the liver	65, 161
miR-99a-5p	Plasma	Human (Asian)	10-16GW	Differential ultracentrifugation	TEM; NTA; WB	↓	mTOR↑, NLRP3↑ in macrophages	65, 162
miR-99b-5p	Placenta	Human (Caucasian/White)	At term	Differential ultracentrifugation+density gradient centrifugation	TEM; NTA; WB	↑	mTOR↓ in the kidney	58, 163
miR-125a-3p	Placenta	Human (Caucasian/White)	At term	Differential ultracentrifugation+density gradient centrifugation	TEM; NTA; WB	↑	Adipogenesis↑	58, 164
miR-197-3p	Placenta	Human (Caucasian/White)	At term	Differential ultracentrifugation+density gradient centrifugation	TEM; NTA; WB	↑	ROBO1↓ in the UVECs	58, 165
miR-22-3p	Placenta	Human (Caucasian/White)	At term	Differential ultracentrifugation+density gradient centrifugation	TEM; NTA; WB	↑	GLUT4↑ in the placenta	58, 166
miR-200-3p	Placenta	Human (Caucasian/White)	At term	Differential ultracentrifugation+density gradient centrifugation	TEM; NTA; WB	↑	TGF-β2/Smad↓ in retina tissue	58, 167
miR-101-3p	Breast milk	Human (Asian)	Lactation	Ultracentrifugation	TEM; NTA; WB	↑	mTOR↓ in hepatocytes	168
miR-222-3p	Plasma	Human (Caucasian/White)	Late gestation	ExoQuick PlExoQuick asma prep and Exosome precipitation kit	WB	↑	ER-α↓, GLUT4↓ in the adipose tissue (when miR-222-3p is upregulated)	62, 78
	Urine (PLAP+)	Human (Caucasian/White)	8-20GW; 24-28GW; 32-39GW	Urine Exosome Purification kit	TEM; WB	8-20GW (-); 24-28GW↑; 32-39GW↓		78
miR-409-3p	Plasma	Human (Caucasian/White)	Late gestation	Plasma prep and Exosome precipitation kit	WB	↑	Positively correlated with FPG and HbA1C	62
has-miR-451a	Placenta	Human (Caucasian/White)	At term	Differential ultracentrifugation+density gradient centrifugation	TEM; NTA; WB	↓	Glycerol kinase**↑** in the liver	58, 169
Let-7c-5p	Plasma	Human (Asian)	8-12GW	Ultracentrifugation	TEM; NTA; WB	↓	Interacts with NRG4-ERBB4 in the liver	60
miR-27a-5p	Plasma	Human (Asian)	8-12GW	Ultracentrifugation	TEM; NTA; WB	↓	PPARγ↑, PI3K/AKT-GLUT4↑ in adipocytes	60
miR-320b	Placenta	Human (Asian)	At term	Differential centrifugation	TEM; NTA; WB	↑	β cell apoptosis↑	64
miR-135a-5p	Plasma (PLAP+)	Human (Asian)	24-28GW	exoEasy Maxi kit	TEM; NTA; WB	↑	Sirtuin 1↑, PI3K/AKT↑ in the trophoblast cells	170
miR-516b-5p	Urine (PLAP+)	Human (Caucasian/White)	8-20GW; 24-28GW; 32-39GW	Urine Exosome Purification kit	TEM; WB	8-20GW (-); 24-28GW↑; 32-39GW↓	Regulates gene expression in the placenta and adipose tissue	78
miR-517-5p	Urine (PLAP+)	Human (Caucasian/White)	8-20GW; 24-28GW; 32-39GW	Urine Exosome Purification kit	TEM; WB	8-20GW (-); 24-28GW↑; 32-39GW↓	Regulates gene expression in the placenta and adipose tissue	78
miR-518a-3p	Urine (PLAP+)	Human (Caucasian/White)	8-20GW; 24-28GW; 32-39GW	Urine Exosome Purification kit	TEM; WB	8-20GW (-); 24-28GW↑; 32-39GW↓	Regulates gene expression in the placenta and adipose tissue	78

ACADM: Acyl-CoA Dehydrogenase Medium Chain; ER-α: Estrogen Receptor-alpha; FGF2: Fibroblast Growth Factor 2; Foxo3: Forkhead box O3; FPG: Fasting Plasma Glucose; GDM: Gestational Diabetes Mellitus; GLUT4: Glucose Transporter Type 4; GW: Gestational Weeks; GYS1: Glycogen Synthase 1; HbA1C: Hemoglobin A1C; IGF1R: Insulin-like Growth Factor 1 Receptor; INSR1: Insulin Receptor Substrate 1; IRS1: Insulin Receptor Substrate 1; IRS2: Insulin Receptor Substrate 2; KLF7: Kruppel-like factor 7; mTOR: Mechanistic Target of Rapamycin; NLRP3: NLR Family Pyrin Domain Containing 3; NOS2: Nitric Oxide Synthase 2; NRG4: Neuregulin 4; NTA: Nanoparticle Tracking Analysis; PLAP: Placental Alkaline Phosphatase; PPARα: Peroxisome Proliferator-Activated Receptor Alpha; PPARγ: Peroxisome Proliferator-Activated Receptor Gamma; PI3K/AKT: Phosphoinositide 3-kinase/Protein Kinase B; ROBO1: Roundabout Guidance Receptor 1; SCD-1: Stearoyl-CoA Desaturase-1; Sirt1: Sirtuin 1; SOCS2: Suppressor of Cytokine Signaling 2; STAT3: Signal Transducer and Activator of Transcription 3; TEM: Transmission Electron Microscopy; TGFB2: Transforming Growth Factor Beta 2; WB: Western Blotting.

**Table 2:** Proteins in exosomes from GDM.

Contents in GDM exosomes	Source	Sample/Model	Trimester of pregnancy	Isolation of exosomes	Characterization of exosomes	Differential expression in GDM	Target/effect	Reference
Complement C3, C5, C4B, C4BPB and C4BPA	Serum	Human (Caucasian/White)	Late gestation	Differential centrifugation	Dynamic light scattering (DLS); Zeta potential; WB	↓	The classical complement/antigen-binding pathway↓	79
C7, C9, F12	Serum	Human (Caucasian/White)	Late gestation	Differential centrifugation	DLS; Zeta potential; WB	↑	The complement terminal pathway and associated inflammatory and coagulation processes↑	79
BANF1	Placenta (PLAP+)	Human (Caucasian/White)	At term	Ultracentrifugation	NTA; WB	↑	cGAS-STING in the placenta↓	83
CARHSP1	Placenta (PLAP+)	Human (Caucasian/White)	At term	Ultracentrifugation	NTA; WB	↓	Gluconeogenesis in the liver↑	83
CRYZL1	Placenta (PLAP+)	Human (Caucasian/White)	At term	Ultracentrifugation	NTA; WB	↑	Affect cell apoptosis and oxidative stress	83
PSG3	Placenta (PLAP+)	Human (Caucasian/White)	At term	Ultracentrifugation	NTA; WB	↑	Regulate insulin secretion	83
ACPP	Placenta (PLAP+)	Human (Caucasian/White)	At term	Ultracentrifugation	NTA; WB	↑	Regulate immunity	83
GBP1	Placenta (PLAP+)	Human (Caucasian/White)	At term	Ultracentrifugation	NTA; WB	↓	Associated with endothelial dysfunction	83
OTUB1	Placenta (PLAP+)	Human (Caucasian/White)	At term	Ultracentrifugation	NTA; WB	↓	Affect cell signal transduction	83
Thbs1	ADSCs	Mice (C57BL/6 J)	12 days of gestation	Differential centrifugation	TEM; NTA; WB	↑	Tgfβ/Smad2 in hepatocytes↑	18
GDF3	Umbilical cord blood	Human (Asian)	24-28GW	Ultracentrifugation	NTA	↑	The adipocytes enlarge	85
PROM1	Umbilical cord blood	Human (Asian)	24-28GW	Ultracentrifugation	NTA	↑	Associated with GDM-related macrosomia	85

ACPP: Acid Phosphatase, Prostate; ADSCs: Adipose-derived stem cells; BANF1: Barrier To Autointegration Factor 1; CARHSP1: Calcium Regulated Heat Stable Protein 1; C4BPA: Complement Component 4 Binding Protein Alpha; C4BPB: Complement Component 4 Binding Protein Beta; CRYZL1: Crystallin Zeta Like 1; F12: Coagulation Factor XII; GDF3: Growth Differentiation Factor 3; GBP1: Guanylate Binding Protein 1; GW: Gestational Weeks; ITIH2: Inter-Alpha-Trypsin Inhibitor Heavy Chain 2; NTA: Nanoparticle Tracking Analysis; PLAP: Placental Alkaline Phosphatase; PROM1: Prominin 1; PSG3: Pregnancy Specific Beta-1-Glycoprotein 3; PTGES3: Prostaglandin E Synthase 3; RANBP1: RAN Binding Protein 1; Thbs1: Thrombospondin 1; TREML2: Triggering Receptor Expressed On Myeloid Cells Like 2; TEM: Transmission Electron Microscopy; Tgfβ: Transforming Growth Factor Beta; WB: Western Blotting.

**Table 3:** Other non-classical contents in exosomes from GDM.

Contents in GDM exosomes	Source	Sample/Model	Trimester of pregnancy	Isolation of exosomes	Characterization of exosomes	Differential expression in GDM	Target/effect	Reference
hsa_circRNA_0039480	Plasma	Human (Asian)	11-13GW; 24-26GW; 36-41GW	Polymer precipitation	TEM; NTA; WB	↑	Positively correlated with OGTT	61
AC006064.4	Umbilical cord blood	Human (Asian)	24-28GW	Ultracentrifugation	NTA	↑	Associated with GDM-related macrosomia	85
lnc-HPS6-1:1	Umbilical cord blood	Human (Asian)	24-28GW	Ultracentrifugation	NTA	↑	Associated with GDM-related macrosomia	85
circ_0014635	Umbilical cord blood	Human (Asian)	24-28GW	Ultracentrifugation	NTA	↑	Associated with GDM-related macrosomia	85
lnc-ZFHX3-7: 1	Umbilical cord blood	Human (Asian)	24-28GW	Ultracentrifugation	NTA	↓	Associated with GDM-related macrosomia	85
*Firmicutes*	Serum	Human (Asian)	24-27GW	Ultracentrifugation	TEM; nFCM	↑	Inflammation↑	89
*Bacteroidetes*	Serum	Human (Asian)	24-27GW	Ultracentrifugation	TEM; nFCM	↓	Butyrate↓	89

GW: Gestational Weeks; nFCM: nano-Flow Cytometry; NTA: Nanoparticle Tracking Analysis; OGTT: Oral Glucose Tolerance Test; TEM: Transmission Electron Microscopy; WB: Western Blotting.

## Abbreviations

ACLY: ATP Citrate Lyase
ADSCs: Adipose-Derived Stem Cells
AKT: Protein Kinase B
BANF-1: Barrier To Autointegration Factor
CARHSP1: Calcium-Regulated Heat-Stable Protein 1
DNMT: DNA Methyltransferase
DOHaD: The Developmental Origins of Health and Disease
EVs: Extracellular Vesicles
GDM: Gestational Diabetes Mellitus
GW: Gestational Weeks
ICAM-1: Intercellular Adhesion Molecule-1
MTTP: Microsomal Triglyceride Transfer Protein
MVBs: Multivesicular Bodies
NAFLD: Non-Alcoholic Fatty Liver Disease
NGT: Normal Glucose Tolerance
NOX4: NADPH Oxidase 4
NTA: Nanoparticle Tracking Analysis
Sirt1: Sirtuin 1
SOCS2: Suppressor of Cytokine Signaling 2
SREBP-1c: Sterol Regulatory Element-Binding Protein-1C
Thbs1: Thrombospondin 1
UVECs: Umbilical Vein Endothelial Cells
